# Supplementary material for: Content-rich biological network constructed by mining PubMed abstracts
Source: BMC Bioinformatics. 2004 Oct 8;5:147. doi: 10.1186/1471-2105-5-147 (PMC528731; doi:10.1186/1471-2105-5-147)
Supplement: Additional File 2 — The original results of the above study (non-essential files are deleted to keep the file size under the limit set by BMC bioinformatics). [file 1471-2105-5-147-S2.bz2 › chilibotAdditionalFile2/dip30/635ID10933786E11022/html/B2M_FCGRT.html]

 


 **B2M** and **FCGRT** 
  
Found 24 abstracts in PubMed, retrieved 24.  
 

 What does Google say? 
 PDF only 
| .edu only 

---

**Interactive relationship** (e.g. stimulation, inhibition, etc)

**Neutral relationship**- The heavy chain of FcRn  [ **FCGRT** ]  was associated with the beta 2 microglobulin  [ **B2M** ]  beta 2 m light chain in U937 and THP 1 cells.  Ref: 11207281 J Immunol, 2001
- Several functions attributed to FcRn  [ **FCGRT** ]  are affected in beta 2 microglobulin  [ **B2M** ]  beta 2 m deficient mice, suggesting that the alpha chain needs to assemble with beta 2 m to form a functional receptor.  Ref: 12006623 J Cell Sci, 2002
- The heavy chain HC of the neonatal Fc receptor  [ **FCGRT** ]  FcRn  [ **FCGRT** ]  for IgG is non convalently associated with beta 2 microglobulin  [ **B2M** ]  beta 2 m.  Ref: 12162790 Biochem J, 2002
- Using multilabeling fluorescence immunohistochemistry and confocal laser scanning microscopy, we found that the neonatal Fc receptor  [ **FCGRT** ]  co localizes with IgG and beta 2 microglobulin  [ **B2M** ]  in granules of human placental syncytiotrophoblast.  Ref: 8766579 Eur J Immunol, 1996
- FcRn  [ **FCGRT** ]  is a heterodimer of an alpha chain and beta 2 microglobulin  [ **B2M** ]  beta 2 m and differs from other IgG Fc receptors in that it is structurally related to MHC class I molecules.  Ref: 12006623 J Cell Sci, 2002
- Functional reconstitution of human FcRn  [ **FCGRT** ]  in Madin Darby canine kidney cells requires co expressed human beta 2 microglobulin  [ **B2M** ] .  Ref: 12023961 J Biol Chem, 2002
- The neonatal IgG transporter FcRn  [ **FCGRT** ]  consists of two chains, FcRn  [ **FCGRT** ]  alpha and beta also known as beta 2 microglobulin  [ **B2M** ] , and is involved in transferring IgG molecules across both mammary and intestinal epithelial cells.  Ref: 12531282 Mol Immunol, 2003
- beta 2 Microglobulin  [ **B2M** ]  is important for cell surface expression and pH dependent IgG binding of human FcRn  [ **FCGRT** ] .  Ref: 12006623 J Cell Sci, 2002
- The major histocompatibility complex class I related neonatal Fc receptor  [ **FCGRT** ] , FcRn  [ **FCGRT** ] , assembles as a heterodimer consisting of a heavy chain and beta 2 microglobulin  [ **B2M** ]  beta 2 m, which is essential for FcRn  [ **FCGRT** ]  function.  Ref: 12023961 J Biol Chem, 2002
- FcRn  [ **FCGRT** ]  was expressed in RPTECs and physically associated with beta 2 microglobulin  [ **B2M** ] , preserving the capacity of specific pH dependent IgG binding.  Ref: 11788451 Am J Physiol Renal Physiol, 2002
- The presence of beta 2 microglobulin  [ **B2M** ]  at the apical plasma membrane corresponds to the recently observed association of beta 2 microglobulin  [ **B2M** ]  with HFE and FcRn  [ **FCGRT** ] .  Ref: 11935295 Histochem Cell Biol, 2002
- Maternal IgG from milk is transported across the intestinal epithelium of neonatal rats by an Fc receptor FcRn  [ **FCGRT** ]  that comprises an alpha chain similar to the class I Ag of the MHC and beta 2 microglobulin  [ **B2M** ] .  Ref: 7504013 J Immunol, 1993
- The MHC class I like FcR, similar to the receptor found in neonatal rodent intestine, FcRn  [ **FCGRT** ] , is present intracellularly in human syncytiotrophoblasts, as is its light chain beta 2 microglobulin  [ **B2M** ] .  Ref: 8944053 APMIS Suppl, 1996
- This receptor FcRn  [ **FCGRT** ] , which helps newborn animals to acquire passive immunity, is an MHC class I like heterodimer made up of a heavy chain and beta 2 microglobulin  [ **B2M** ] .  Ref: 7538537 J Immunol, 1995
- The rat intestinal Fc receptor FcRn  [ **FCGRT** ]  consists of a heavy chain, with significant sequence similarity to the heavy chain of class I MHC molecules, complexed to the class I light chain, beta 2 microglobulin  [ **B2M** ] .  Ref: 8478919 J Mol Biol, 1993

**Non-interactive relationship** (e.g. studied together, co-existance, homology, etc.)

- The heavy chain of FcRn  [ **FCGRT** ]  has sequence similarity in three extracellular domains to the corresponding domains of class I molecules, and the light chain of both types of molecules is beta 2 microglobulin  [ **B2M** ] .  Ref: 1530991 Proc Natl Acad Sci U S A, 1992
- beta 2 microglobulin  [ **B2M** ]  mice lack functional molecules of the MHC class I related Fc receptor, FcRn  [ **FCGRT** ] .  Ref: 9014824 Immunology, 1996
- The first marsupial homologues of the Fc receptor heavy and light chains, FcRn  [ **FCGRT** ]  and beta 2 microglobulin  [ **B2M** ] , from the brushtail possum have been cloned and characterised.  Ref: 11090878 Mol Immunol, 2000
- The non covalent association of beta 2 microglobulin  [ **B2M** ]  with MHC class I molecules and MHC class I type molecules such as FcRn  [ **FCGRT** ]  or the hemochromatosis protein HFE is of major importance for their function, i.e., antigen presentation, IgG transport, and regulation of iron uptake, respectively.  Ref: 11935295 Histochem Cell Biol, 2002
- Using in situ hybridisation, the FcRn  [ **FCGRT** ]  alpha and beta 2 microglobulin  [ **B2M** ]  transcripts were localised to the epithelial cells of the PY intestine.  Ref: 12531282 Mol Immunol, 2003
- Increased clearance of IgG in mice that lack beta 2 microglobulin  [ **B2M** ]  possible protective role of FcRn  [ **FCGRT** ] .  Ref: 9014824 Immunology, 1996
- The results are consistent with the hypothesis that changes in FcRn  [ **FCGRT** ]  alpha and beta 2 microglobulin  [ **B2M** ]  gene expression take place in the possum PY intestine to accommodate changes in maternal milk composition to meet the changing immunological demands of the PY.  Ref: 12531282 Mol Immunol, 2003
- Absence of TPKTSVT placental homing in mice lacking beta 2 microglobulin  [ **B2M** ]  beta 2 m suggests FcRn  [ **FCGRT** ]  beta 2 m as a target for the TPKTSVT, which is unexpected, given the normal development of FcRn  [ **FCGRT** ]  beta 2 m deficient progeny.  Ref: 12242328 Proc Natl Acad Sci U S A, 2002
- This RNA contained functional FcRn  [ **FCGRT** ]  transcripts as it encoded a beta 2 microglobulin  [ **B2M** ]  associated cell surface protein as determined by immunoprecipitation of biotinylated cell surface proteins with a polyclonal anti FcRn  [ **FCGRT** ]  specific antiserum.  Ref: 7738203 J Clin Invest, 1995
- However, no prolactin receptor transcripts were detected in the same epithelial cells suggesting that the observed changes in FcRn  [ **FCGRT** ]  alpha and beta 2 microglobulin  [ **B2M** ]  gene expression in the proximal intestine are not modulated directly by prolactin.  Ref: 12531282 Mol Immunol, 2003
- Genomic sequences located 5 to the start of transcription of the FcRn  [ **FCGRT** ]  alpha and beta 2 microglobulin  [ **B2M** ]  genes were cloned and analysed for predicted cis acting transcription control elements.  Ref: 12531282 Mol Immunol, 2003
- To determine whether the slower degradation of immunoglobulin in mice with beta 2 microglobulin  [ **B2M** ]  correlated with the ability of the antibody to bind FcRn  [ **FCGRT** ] , we measured the clearance of chicken IgY, which does not bind this receptor.  Ref: 9014824 Immunology, 1996
- Both the FcRn  [ **FCGRT** ]  alpha and beta 2 microglobulin  [ **B2M** ]  genomic sequences contained STAT5 binding motifs consistent with the transcription of both genes being modulated by prolactin.  Ref: 12531282 Mol Immunol, 2003
